# A Superhydrophilic Aluminum Surface with Fast Water Evaporation Based on Anodic Alumina Bundle Structures via Anodizing in Pyrophosphoric Acid

**DOI:** 10.3390/ma12213497

**Published:** 2019-10-25

**Authors:** Daiki Nakajima, Tatsuya Kikuchi, Taiki Yoshioka, Hisayoshi Matsushima, Mikito Ueda, Ryosuke O. Suzuki, Shungo Natsui

**Affiliations:** 1 Faculty of Engineering, Hokkaido University, N13-W8, Kita-ku, Sapporo, Hokkaido 060-8628, Japan; d.nakajima@eng.hokudai.ac.jp (D.N.); tysok0520@gmail.com (T.Y.); matsushima@eng.hokudai.ac.jp (H.M.); mikito@eng.hokudai.ac.jp (M.U.); rsuzuki@eng.hokudai.ac.jp (R.O.S.); 2 Institute of Multidisciplinary Research for Advanced Materials, Tohoku University, Katahira 2-1-1, Aoba-ku, Sendai, Miyagi 980-8577, Japan; shungo.natsui.b7@tohoku.ac.jp

**Keywords:** aluminum, anodizing, pyrophosphoric acid, superhydrophilicity, alumina nanofiber

## Abstract

A superhydrophilic aluminum surface with fast water evaporation based on nanostructured aluminum oxide was fabricated via anodizing in pyrophosphoric acid. Anodizing aluminum in pyrophosphoric acid caused the successive formation of a barrier oxide film, a porous oxide film, pyramidal bundle structures with alumina nanofibers, and completely bent nanofibers. During the water contact angle measurements at 1 s after the water droplet was placed on the anodized surface, the contact angle rapidly decreased to less than 10°, and superhydrophilic behavior with the lowest contact angle measuring 2.0° was exhibited on the surface covered with the pyramidal bundle structures. As the measurement time of the contact angle decreased to 200–33 ms after the water placement, although the contact angle slightly increased in the initial stage due to the formation of porous alumina, at 33 ms after the water placement, the contact angle was 9.8°, indicating that superhydrophilicity with fast water evaporation was successfully obtained on the surface covered with the pyramidal bundle structures. We found that the shape of the pyramidal bundle structures was maintained in water without separation by in situ high-speed atomic force microscopy measurements.

## 1. Introduction

Aluminum possesses many attractive mechanical and physical properties, such as high strength-to-weight ratio, heat conductivity, electrical conductivity, and reflectivity; thus, aluminum alloys have been used for various industrial applications. Recently, wettability control on aluminum surfaces has been an important strategy for corrosion protection, heat exchange devices, and anti-icing surfaces [1,2,3,4,5,6,7,8,9,10,11,12,13]. For example, a superhydrophilic aluminum surface with a water contact angle (*θ*_WCA_) measuring less than 10° exhibits rapid spreading of a water droplet, and such aluminum substrates have potential as high-efficiency plate-fin heat exchangers and antifouling materials [14,15]. Conversely, superhydrophobic aluminum surfaces measuring more than *θ*_WCA_ = 150° with a low contact angle hysteresis exhibit excellent water-sliding behavior and have potential for self-cleaning materials and highly corrosion-resistant surfaces. Therefore, many surface finishing processes including painting [14], plasma treatment [16,17], colloidal coating [18], and carbon nanoparticle deposition [19] have been widely investigated for the fabrication of superhydrophilic and superhydrophobic aluminum surfaces.

Electrochemical anodizing is one of the most important surface finishing processes for aluminum and its alloys and is widely employed for dielectric film coating [20,21,22], coloring [23,24,25], hardening [26,27,28], corrosion resistance improvements [29,30,31], and novel nanomaterial fabrication [32,33,34,35,36,37,38,39,40,41,42,43]. So far, processes combining traditional anodizing in sulfuric, oxalic, and phosphoric acids with self-assembled monolayer (SAM) coatings have been reported by several research groups for superhydrophilic and superhydrophobic aluminum surfaces [44,45]. In this technique, anodic alumina nanofibers are formed on the surface via long-term anodizing and chemical dissolution of the upper part of porous alumina, and their nanostructure exhibits superhydrophilic behavior. In addition, subsequent hydrophobic SAM modification causes superhydrophobicity on the surface. However, it is difficult to accurately control the morphology of alumina nanofibers during anodizing due to the uneven chemical dissolution. Very recently, we have found the formation of alumina nanofibers formed by anodizing in a novel electrolyte, pyrophosphoric acid. Pyrophosphoric acid anodizing improves the controllability of the morphology of the alumina nanofibers due to the rapid dissolution of the anodic oxide and subsequent growth of the nanofibers during the initial stage of anodizing. Thus, highly ordered nanofiber arrays can be easily fabricated on the aluminum surface [46,47,48]. Superhydrophobic aluminum surfaces are successfully fabricated by anodizing in pyrophosphoric acid and with SAM modification [49]. Moreover, the fabrication of highly slippery and sticky superhydrophobic aluminum surfaces is easily achieved via the nanostructure control of alumina nanofibers [50]. On the other hand, the nanofiber-covered aluminum surface without SAM modification exhibits superhydrophilic behavior [51,52,53]. The contact angle measured on the superhydrophilic aluminum surface greatly changed with the anodizing time. In addition, it was observed that water evaporated quickly from the superhydrophilic surface. However, the effect of the morphology of the alumina nanofibers on the superhydrophilicity and corresponding fast water evaporation is still unclear.

In the present investigation, we describe the superhydrophilicity on the aluminum surfaces covered with anodic alumina nanofibers and subsequent alumina bundle structures by anodizing in pyrophosphoric acid under the optimum operating condition. The superhydrophilic behavior was investigated by water contact angle investigation using a high-speed camera and water evaporation rate measurements. The morphology of the alumina nanofibers was examined by scanning electron microscopy and in situ high-speed atomic force microscopy, and a contact angle measuring less than 10° within 33 ms indicated that superhydrophilicity with fast water evaporation was successfully achieved by the formation of pyramidal alumina bundle structures on the surface.

## 2. Experimental

First, 99.99 wt % aluminum plates (thickness: 400 μm, Nippon Light Metal, Tokyo, Japan) were cut into rectangular pieces (width: 20 mm × height: 40 mm), which then were degreased in ethanol for 10 min using an ultrasonic cleaner (US-1R, AS ONE, Osaka, Japan) The specimens were electrochemically polished in 22 vol% 70%-HClO_4_/78 vol% CH_3_COOH solution at 280 K and a constant voltage of 28 V for 1 min.

The specimens were anodized in a 74.0% pyrophosphoric acid solution (Kanto Chemical, Tokyo, Japan) under constant voltage conditions for the fabrication of anodic alumina nanofibers. During this process, an electrochemical cell with an inner diameter of 55 mm was filled with 100 mL pyrophosphoric acid solution, and the aluminum specimen as an anode and a platinum plate as a cathode (99.95 wt %, width: 16 mm × height: 28 mm × thickness: 100 μm, Furuya Metal, Tokyo, Japan) were immersed in the electrolyte solution. The distance of the aluminum anode and the platinum cathode was adjusted to 20 mm in the electrochemical cell. The temperature of the electrolyte solution was maintained at 283 K in a large-scale water bath (UCT-1000, AS ONE), and the pyrophosphoric acid was stirred at a rate of 1 s^−1^ with a magnetic stirrer (MS-101, AS ONE). The aluminum specimens were anodized at constant cell voltages of V = 75–80 V for up to 180 min using a DC power supply (PWR-400H, Kikusui, Yokohama, Japan). This experimental condition was chosen for the formation of typical anodic alumina nanofibers during pyrophosphoric acid anodizing [47]. After the electrochemical process, the specimens were quickly removed from the electrolyte solution and then washed with ultrapure water. Some anodized specimens were immersed in a 0.52 M phosphoric acid solution at 293 K for up to 20 min to dissolve the anodic oxide.

The nanostructured surface of the specimen was characterized by field-emission scanning electron microscopy (FE-SEM, JSM-6500F, JEOL, Akishima, Japan) and atomic force microscopy (AFM, Nanocute, Hitachi High-Technologies, Tokyo, Japan). Before SEM observations, the anodized specimens were covered with a thin platinum electroconductive layer by sputter coating. The nanostructure of the anodic oxide was quantified with a computerized image analysis software (Image-Pro, Media Cybernetics, Rockville, MD, USA). The anodized specimens were also examined by high-speed AFM (HS-AFM) with an imaging rate of 0.5 frames per second (fps) for in situ observation of anodic alumina nanofibers in ultrapure water.

The contact angles of an ultrapure water droplet (specific resistance: 18.2 MΩ·cm) formed on the aluminum specimen were measured by an optical contact angle meter (DM-501, Kyowa Interface Science, Niiza, Japan). The water droplet adjusted to 0.5 μL in volume was placed on the surface of the anodized specimens, and the water contact angles were continuously measured from 33 ms to 1 s just after the water droplet was placed on the anodized surface. The contact angle investigations were made at five different surfaces, and the obtained contact angles were averaged without the maximum and minimum contact angles. Evaporation behavior of the water droplet was also investigated on the anodized aluminum surface. A 0.5 μL water droplet was placed on the anodized specimens, and the time required for the complete evaporation of the water droplet from the surface was measured by a charge-coupled device (CCD) camera. During evaporation measurements, the temperature of the anodized specimens was maintained at 313 K using a thermoelectric Peltier module (CP-085, Scinics, Tokyo, Japan). The environment temperature and humidity were not controlled.

## 3. Results and Discussion

**Figure 1:**
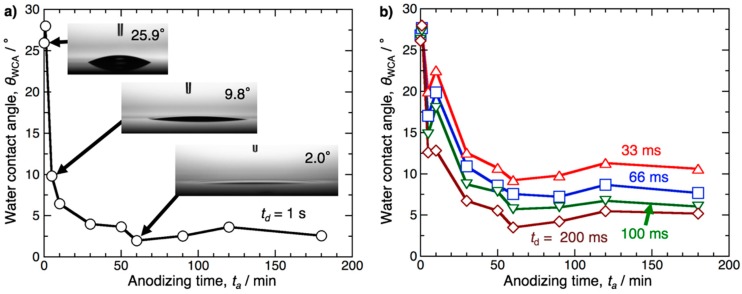
(**a**) Change in the water contact angle measured on the anodized aluminum surface, *θ*_WCA_, with the anodizing time *t*_a_. The electropolished specimens were anodized in pyrophosphoric acid at 283 K and 80 V for 1−180 min, and the contact angles were measured at *t*_d_ = 1 s after a 0.5 μL water droplet was placed on the surface. (**b**) The *θ*_WCA_ − *t*_a_ curves obtained at *t*_d_ = 200, 100, 66, and 33 ms.

The pretreatment specimens were anodized in pyrophosphoric acid at 80 V and 283 K for up to 180 min. Figure 1a shows the change in the water contact angle measured on the aluminum specimen, *θ*_WCA_, with the anodizing time, *t*_a_. Here, the *θ*_WCA_ values were measured at *t*_d_ = 1 s after the 0.5 μL water droplet was placed on the surface. The electropolished aluminum surface exhibited hydrophilicity measuring *θ*_WCA_ = 25.9° due to the formation of a hydrophilic, thin native oxide film after electropolishing [54]. As the electropolished specimen was anodized for 5 min, a superhydrophilic surface measuring *θ*_WCA_ = 9.8° was successfully obtained due to the formation of anodic aluminum oxide. The *θ*_WCA_ then gradually decreased with increasing anodizing time, and lower contact angles measuring 2°–4° were obtained after anodizing for 30–180 min. Notably, the minimum contact angle measuring 2.0° was obtained on the surface anodized for 60 min, and it appears that the contact angle very slightly increased with the anodizing time, although it is difficult to conclude a significant difference based on these small values. Therefore, the contact angles were also measured at the early stages after the droplet was placed on the surface using a high-speed CCD camera.

Figure 1b shows the *θ*_WCA_ − *t*_a_ curves obtained at *t*_d_ = 200, 100, 66, and 33 ms after the 0.5 μL water droplet was placed on the surface. Although similar tendencies were obtained at *t*_d_ = 200 ms through 33 ms, the contact angles obtained at each anodizing time decreased with increasing *t*_d_ value. The minimum contact angle was measured at *t*_a_ = 60 min in either case except for 66 ms, and superhydrophilicity, with a contact angle measuring *θ*_WCA_ = 9.2°, was exhibited on the surface anodized for *t*_a_ = 60 min within only 33 ms after the water placement.

**Figure 2:**
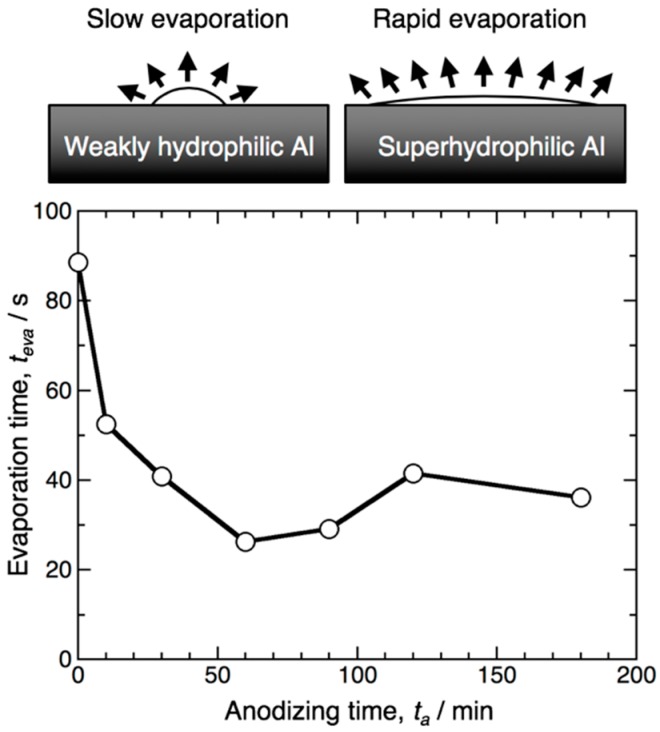
Change in the evaporation time of a 0.5 μL water droplet, *t*_eva_, with the anodizing time, *t*_a_, on the aluminum specimen anodized at 283 K and 80 V. The temperature of the aluminum substrate was maintained at 313 K using a thermoelectric Peltier module.

As the water droplet was placed on the superhydrophilic surface, the droplet spread isotropically and soon evaporated from the surface. Because the evaporation time of the water droplet strongly depends on the hydrophilicity of the surface, the evaporation time was investigated on the aluminum surface anodized for various operating times. Figure 2 shows the change in the evaporation time of a 0.5 μL water droplet on the specimen anodized under the same operating conditions shown in Figure 1, *t*_eva_, with the anodizing time, *t*_a_. Here, the temperature of the aluminum substrate was maintained at 313 K using a thermoelectric Peltier module during the measurements. The time required for the complete evaporation of the water droplet from the electropolished surface was measured to be *t*_eva_ = 88.5 s. The evaporation time decreased with the increased anodizing time, and the minimum rapid evaporation time measuring *t*_eva_ = 26.3 s was obtained on the surface anodized for *t*_a_ = 60 min. However, excess anodizing for more than 90 min caused the evaporation time increase to *t*_eva_ = 29.2–41.6 s. Comparing Figure 2 with Figure 1, the shape of this *t*_eva_ − *t*_a_ curve is in good agreement with the *θ*_WCA_ − *t*_a_ curves shown in Figure 1. It is clear from Figure 1 and Figure 2 that the highest superhydrophilicity can be obtained on the aluminum surface anodized for 60 min. To investigate the effect of the morphology of the anodic oxide formed by pyrophosphoric acid anodizing on the superhydrophilic behavior, the surface of the anodized specimens was characterized by SEM.

**Figure 3:**
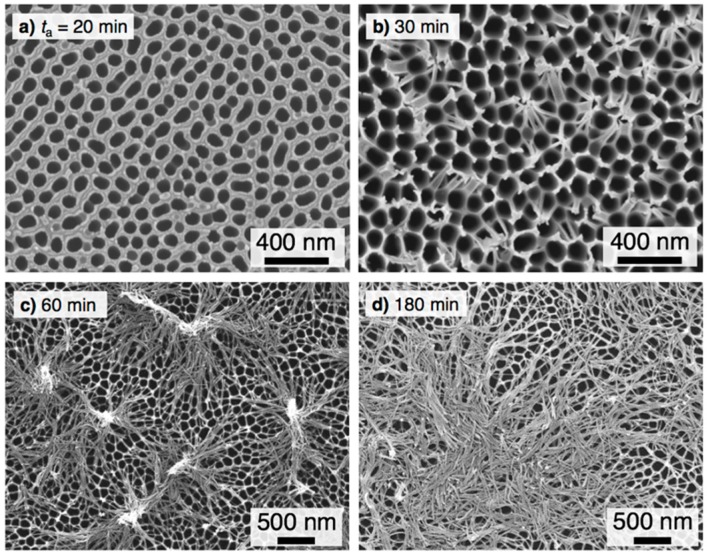
SEM (scanning electron microscopy) images of the aluminum specimen anodized at 283 K and 80 V. *t*_a_ = (**a**) 20 min, (**b**) 30 min, (**c**) 60 min, (**d**) 180 min.

Figure 3 shows SEM images of the aluminum specimen anodized in pyrophosphoric acid at 80 V and 283 K for (a) 20 min, (b) 30 min, (c) 60 min, and (d) 180 min. As the electropolished aluminum specimen was anodized for 20 min (Figure 3a), a porous oxide film with numerous pores measuring 87 nm in average pore diameter was formed on the aluminum surface. Increasing the anodizing time to 30 min (Figure 3b) led to the growth of alumina nanofibers at the triple points of the honeycomb structure due to the chemical dissolution of anodic oxide during anodizing, and small pyramidal bundle structures consisting of several nanofibers were formed on the surface because of the bending and subsequent tangling of the alumina nanofibers. The number of alumina nanofibers contained in each bundle structure increased with the anodizing time due to the growth of the alumina nanofibers, and larger bundle structures were formed by anodizing for 60 min (Figure 3c). Further anodizing caused the formation of longer alumina nanofibers and subsequent complete bending due to their own weight, and the surface was covered with the bent alumina nanofibers (Figure 3d). Comparing the SEM images with the contact angle measurements and the evaporation behaviors, the following results are obtained: (a) Superhydrophilicity can be obtained on the porous alumina- and nanofiber-covered aluminum surface formed by anodizing in pyrophosphoric acid. (b) In particular, the highest superhydrophilicity is exhibited on the surface covered with the large pyramidal bundle structures after anodizing for 60 min.

**Figure 4:**
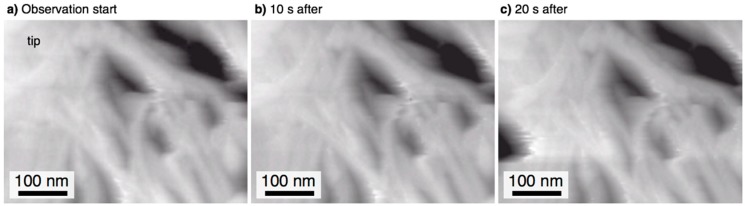
In situ HS-AFM (high-speed atomic force microscopy) images of the pyramidal bundle structure consisting of anodic alumina nanofibers in ultrapure water: (**a**) when observation starts, (**b**) 10 s after, and (**c**) 20 s after. The aluminum specimen anodized at 293 K and 75 V for 15 min.

When the water droplet is placed on the aluminum surface covered with the pyramidal alumina bundle structures, one question is whether or not the shape of the bundles is maintained in water. To reveal the morphology of the alumina bundle structures in ultrapure water, the specimen anodized for 60 min was examined by in situ HS-AFM. Figure 4a shows a HS-AFM image of a bundle structure formed by anodizing in pyrophosphoric acid at 293 K and 75 V for 15 min. It is clear that the bundle structures consisting of many alumina nanofibers maintained their shape in ultrapure water; the alumina nanofibers were not separated and fluttered in the underwater conditions. Figure 4b,c show HS-AFM images of the bundle structure at 10 s and 20 s after starting the observation, respectively. Although several nanofibers were slightly moved by the scanning of the cantilever, the shape of the bundle structure and the alumina nanofibers was largely maintained during in situ HS-AFM observation for 20 s. Therefore, it is expected that the bundle structures maintained their shapes in water when the water droplet was placed on the anodized surface during the contact angle measurements. The reason that the bundle structures were unchanged in water may be due to the van der Waals forces between the alumina nanofibers [55].

**Figure 5:**
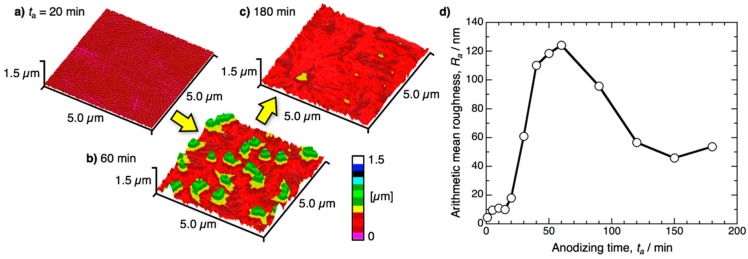
Three-dimensional AFM images of the pyramidal bundle structure consisting of anodic alumina nanofibers formed at 283 K and 80 V. *t*_a_ = (**a**) 20 min, (**b**) 60 min, (**c**) 180 min. (**d**) Change in the arithmetic mean roughness of the anodized specimen, R_a_, with the anodizing time, *t*_a_.

Figure 5a–c depict three-dimensional AFM images of a specimen anodized in pyrophosphoric acid at 283 K and 80 V for (a) *t*_a_ = 20 min, (b) 60 min, and (c) 180 min, respectively. As the aluminum specimen was anodized for 20 min, a porous oxide film was formed on the surface (Figure 3a), and a relatively flat surface with numerous small nanopores was observed in the AFM image (Figure 5a). As the anodizing time increased to 60 min, many convex bundle structures of approximately 1 μm in maximum height were observed to be distributed on the surface (Figure 3c and Figure 5b). However, further anodizing for 180 min caused the disappearance of the bundle structures due to the complete bending of longer alumina nanofibers, and the surface roughness decreased (Figure 3d and Figure 5c). Figure 5d summarizes the change in the arithmetic mean roughness measured from the AFM image, Ra, with the anodizing time. The roughness rapidly increased with the anodizing time during the initial stage due to the formation of alumina nanofibers and subsequent bundle structures, and a maximum roughness measuring Ra = 124 nm was obtained by anodizing for 60 min. With this anodizing time, the highest superhydrophilicity was exhibited on the aluminum surface (Figure 1 and Figure 2). The roughness, then, gradually decreased to approximately 50 nm due to the bending of alumina nanofibers by excess anodizing. The water contact angle measured at this stage increased with the anodizing time. As compared the contact angle investigations with the surface roughness, the water contact angle and hydrophilic behavior clearly depend on the surface roughness.

The contact angle of the droplet formed on the rough surface can be described by Wenzel’s equation as [56]

cos *θ*_W_ = *R* cos *θ* (1)

where *R* corresponds to the specific surface area, and *θ*_W_ and *θ* are the contact angles obtained on the rough surface and the flat surface, respectively. The anodic alumina nanofibers formed by pyrophosphoric acid anodizing consists of pure aluminum oxide without any electrolyte anion, and anodic aluminum oxide exhibits hydrophilicity due to the presence of the surface-bound hydroxyl groups [51]. Therefore, Wenzel’s equation indicates that the contact angle decreases with increasing specific surface area on such a hydrophilic surface. The aluminum surface covered with the pyramidal bundle structures consisting of numerous alumina nanofibers possesses a higher surface roughness (Figure 5d). In addition, SEM observation (Figure 3c) shows that many nanoscale spaces are formed under the pyramidal bundle structures. Moreover, strong capillary forces are induced by these alumina nanofibers. Therefore, the highest superhydrophilicity may be obtained on the surface covered with the pyramidal bundle structures by anodizing for 60 min (Figure 1 and Figure 2). On the other hand, long-term anodizing leads to the disappearance of the bundle structures due to the complete bending of the long alumina nanofibers, and most of the aluminum surface is covered with the bent nanofibers (Figure 3d and Figure 5c). Thus, the contact angle may slightly increase by excess anodizing for more than 60 min (Figure 1 and Figure 2).

**Figure 6:**
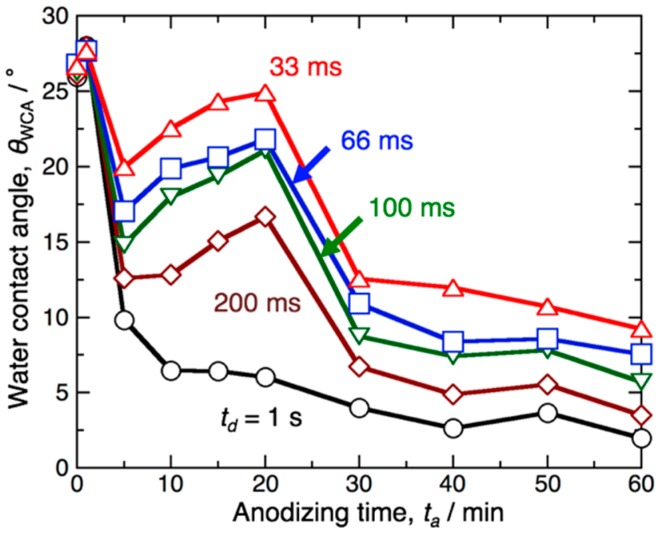
Change in the water contact angle measured on the anodized aluminum surface, *θ*_WCA_, with the anodizing time, *t*_a_, during the initial stage of anodizing. The electropolished specimens were anodized in pyrophosphoric acid at 283 K and 80 V.

Summarizing so far, the pyramidal alumina bundles are important nanostructures for the fabrication of superhydrophilic surfaces with fast water evaporation, but excess anodizing causes the hydrophilicity to decrease due to the disappearance of the bundle structures. On the other hand, short-term anodizing for 10 min caused a slight increase in the contact angle during the high-speed contact angle measurements for *t*_d_ = 33–200 ms (Figure 1b), although the anodic oxide grew on the surface. Therefore, we demonstrate further contact angle investigations in the initial stage of pyrophosphoric acid anodizing in detail. Figure 6 shows the *θ*_WCA_ − *t*_a_ curves for the initial stage of anodizing on the aluminum surface anodized at 80 V and 283 K. At *t*_d_ = 1 s after the droplet was placed on the surface, the contact angle rapidly decreased to *θ*_WCA_ = 9.8° as the specimen was anodized for *t*_a_ = 5 min and then slightly decreased with the anodizing time. However, very little change in the contact angle was measured during the range of 10 min < *t*_a_ < 20 min. Based on the high-speed contact angle investigations at *t*_d_ = 33–200 ms, it is noteworthy that the contact angle gradually increased with the anodizing time from 10 min < *t*_a_ < 20 min, although hydrophilic aluminum oxide was formed on the surface via anodizing. There is a clear difference in the *θ*_WCA_ − *t*_a_ curves between *t*_d_ = 1 s and 200–33 ms.

**Figure 7:**
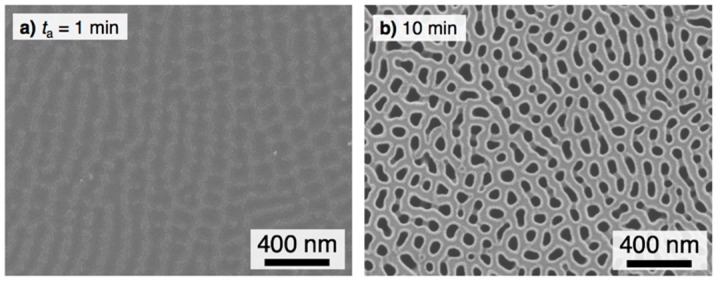
SEM images of the aluminum specimen anodized at 283 K and 80 V (**a**) *t*_a_ = 1 min and (**b**) 10 min.

Figure 7 shows SEM images of the anodized aluminum surface in the initial stage of (a) 1 min and (b) 10 min. A thin barrier oxide film with narrow stripes approximately 100–150 nm wide was observed on the surface anodized for 1 min (Figure 7a). This stripe pattern corresponds to the nanomorphology of the electropolished aluminum surface [57]. As the anodizing time increased to *t*_a_ = 10 min, a porous alumina film with numerous nanopores measuring 52 nm in average diameter was formed (Figure 7b). Further anodizing for *t*_a_ = 20 min caused the pore diameter increase by chemical dissolution of the anodic oxide (average pore diameter: 87 nm, Figure 3a). Therefore, the period of the contact angle increase, *t*_a_ = 10–20 min, corresponds to the expansion of the nanopores in the porous alumina matrix.

**Figure 8:**
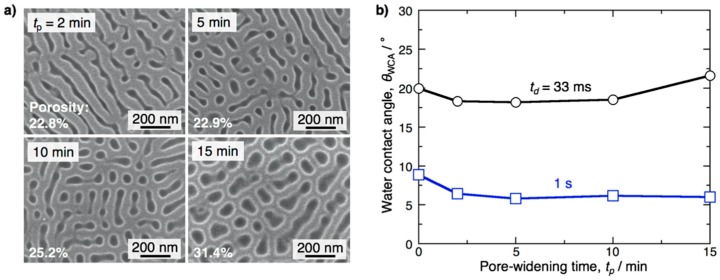
(**a**) SEM images of the porous alumina film formed by pyrophosphoric acid anodizing after pore-widening for *t*_p_ = 2–15 min. (**b**) Changes in the water contact angle, *θ*_WCA_, with the pore-widening time, *t*_p_, at *t*_d_ = 33 ms and 1 s after the water droplet was placed on the surface.

To understand the effect of pore diameter in the porous alumina film on the contact angle, contact angle measurements were also performed using pore-widening porous alumina specimens. Here, the electropolished aluminum specimens were anodized in pyrophosphoric acid at 283 K and 80 V for 5 min to form a porous alumina film, and the anodized specimens were then immersed in a 0.52 M phosphoric acid solution at 293 K for up to *t*_p_ = 15 min for pore-widening. Figure 8a shows SEM images of the surface of the anodized specimen after pore-widening. Circular pores and linear trenches were formed on the surface by pore-widening for 2 min, and the porosity was calculated to be 22.8% using image analysis software. Although the porosity of the porous alumina was almost unchanged in a short immersion time for *t*_p_ = 5 min, it gradually increased with immersion time by long-term pore-widening (25.2% for 10 min and 31.4% for 15 min). The porous alumina film was completely dissolved into the solution by immersion for 20 min. Figure 8b shows the *θ*_WCA_ − *t*_p_ curves obtained on the anodized specimens after pore-widening. At *t*_d_ = 1 s, the contact angle slightly decreased during the initial stage of pore-widening and then was almost unchanged during pore-widening. Conversely, it is clear that the contact angle obtained at *t*_d_ = 33 ms gradually increased with the pore-widening time and corresponding the porosity. Based on Figure 6 and Figure 8, the porosity—in other words, the diameter of the pores formed in the porous alumina film—strongly affects the contact angle obtained at the initial stage after the droplet is placed on the surface, and the contact angle increases with the pore diameter.

**Figure 9:**
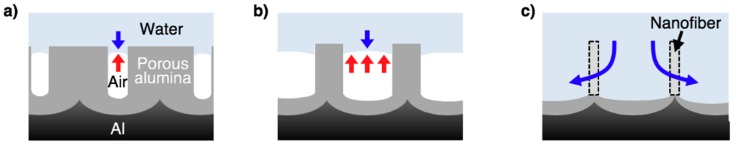
Schematic illustrations of the cross-section of the anodized specimen after the water droplet was placed on the surface: (**a**) porous alumina with narrow pores, (**b**) porous alumina with large-scale pores, and (**c**) nanofiber-covered surface.

When the water droplet is placed on the porous alumina film, water enters into the nanopores of the hydrophilic aluminum oxide. However, because a repulsive force is generated by the air in the pores, a composite surface consisting of aluminum oxide and air may be formed on the surface in the initial stage after the water placement (Figure 9a). The Cassie–Baxter model is widely employed to consider the contact angle formed on such a composite surface, and the contact angle obtained on the solid–air composite surface, *θ*_c_, can be described by the equation [56]

cos *θ*_c_ = *f* (1 + cos *θ*) − 1 (2)

where *f* is the area fraction of the projected contact area and *θ* is the contact angle obtained on the flat surface. This equation indicates that a decrease in the solid area leads to an increase in the contact angle. The reason why the *θ*_WCA_ obtained at *t*_d_ = 33–200 ms increases with the anodizing time in the initial anodizing stage of *t*_a_ = 10–20 min (Figure 6) may be due to this relation. The contact angle increases with the anodizing time for up to 20 min because the diameter of the nanopores increases by pore-widening (Figure 9b). However, water enters into the nanopores of the hydrophilic aluminum oxide as the *t*_d_ value increases, and the composite surface disappears from the surface. Thus, the contact angle slightly decreases with the anodizing time at *t*_d_ = 1 s. On the other hand, the contact angles obtained at whole *t*_d_ values decreased after anodizing for 30 min (Figure 6). As described in Figure 3, anodic alumina nanofibers and subsequent bundle structures were formed on the surface by anodizing for 30 min. The water droplet can quickly spread on the nanofiber-covered surface without closed nanopores due to the capillary effect of the nanofibers (Figure 9c); thus, superhydrophilicity with fast water evaporation appears on the surface after the formation of alumina nanofibers. By in situ HS-AFM, the shape of the pyramidal bundle structures was maintained in water without separation.

Although several research groups have reported that superhydrophilic aluminum could be fabricated by anodizing, the time after the water droplet was placed on the surface during contact angle investigations was unknown in many cases. Ye et al. reported that ‘bird’s nest’ surface fabricated by typical anodizing in phosphoric acid exhibited superhydrophilicity measuring *θ*_WCA_ = 3° when a water droplet was dropped on the surface after around 0.8 s [58]. In our investigation, the minimum contact angles measuring *θ*_WCA_ = 2.0° at *t*_d_ = 1 s and 3.0° at *t*_d_ = 500 ms were obtained on the surface anodized in pyrophosphoric acid for 60 min, and this result is lower than that obtained by the previous investigation. Therefore, our anodizing technique using pyrophosphoric acid is useful for the fabrication of superhydrophilic aluminum surface in the various engineering fields.

## 4. Conclusions

We investigated the superhydrophilicity of an aluminum surface anodized in pyrophosphoric acid. Pyrophosphoric acid anodizing leads to the fabrication of thin uniform oxide, porous oxide, pyramidal bundle structures consisting of many alumina nanofibers, and completely bent nanofibers. The change in the water contact angle with the anodizing time exhibits different behaviors depending on the measurement time after the water droplet is placed on the surface. During the measurement at 1 s after the water placement, the water contact angle greatly decreases to less than *θ*_WCA_ = 10° by pyrophosphoric acid anodizing for up to 5 min, and a superhydrophilic aluminum surface is easily obtained. On the other hand, the contact angle slightly increases in the initial stage of anodizing due to the formation of porous alumina during the measurement at 200–33 ms after the water placement, and then, superhydrophilic behavior is exhibited on the nanofiber-covered surface. In particular, the value *θ*_WCA_ = 9.8° at 33 ms after the water placement indicates that superhydrophilicity with fast water evaporation can be successfully achieved on the surface covered with the pyramidal alumina bundle structures.
